# Impact of consultation-based hospice palliative care team on self-determination respect rates

**DOI:** 10.1017/S1478951525100916

**Published:** 2025-11-03

**Authors:** Hea Lim Choi, Jeong Ah Kim, Mi Hyeon Seo, Eun Jeong Lee, Yu Jeong Heo, Kyung Won Kim, Yoona Lee, In Young Cho, Sang Eun Yoon, Dong Wook Shin

**Affiliations:** 1 Department of Clinical Research Design and Evaluation, Samsung Advanced Institute of Health Science and Technology (SAIHST), Sungkyunkwan University, Seoul, Korea; 2 Center for Trend Sensing-Risk Modeling, Samsung Medical Center, Sungkyunkwan University School of Medicine, Seoul, South Korea; 3 Hospice Palliative Care Team, Samsung Medical Center, Sungkyunkwan University School of Medicine, Seoul, Korea; 4 Department of Family Medicine/Supportive Care Center, Samsung Medical Center, Sungkyunkwan University School of Medicine, Seoul, Korea; 5 Division of Hematology-Oncology, Department of Medicine, Samsung Medical Center, Sungkyunkwan University School of Medicine, Seoul, Korea

**Keywords:** Hospice, palliative care, advanced care planning, palliative care team, self-determination

## Abstract

**Objectives:**

Despite the increasing implementation of consultation-based hospice palliative care teams in tertiary hospitals of Korea, there is limited research on their impact on self-determination respect rates. Understanding this impact is crucial for improving end-of-life care practices and respecting patient autonomy. The aim of this study is to assess the trends in self-determination respect rates regarding advance care planning before and after the introduction of a consultation-based hospice palliative care team in a tertiary hospital.

**Methods:**

A retrospective observational study was conducted using medical records from a tertiary hospital in Korea from March 2018 to December 2023. The study included all patients aged 19 years and older with medical records at a tertiary hospital during the specified period. We examined the characteristics of patients referred to the palliative care team, the effects of the consultation-based hospice palliative care team on the completion rates of advanced care planning, and changes in self-determination respect rates.

**Results:**

Following the introduction of the consultation-based hospice palliative care team, 411 patients were referred. The proportion of patients with completed advance care planning increased from 27.0% to 60.6% (*p* < 0.001). The overall number of advanced care planning completions and the self-determination respect rate also showed a marked increase, particularly from 2021 to 2022, when the respect rate spiked from 27.6% to 43.2%.

**Significance of Results:**

Introduction of a consultation-based hospice palliative care team improved the respect for patient self-determination in end-of-life care decisions. These findings support the integration of hospice care teams in tertiary hospitals to enhance early and informed end-of-life decision-making.

## Introduction

Since the enactment of the Life-Sustaining Treatment Decision-making Act in February 2016 and its enforcement in February 2018, hospice palliative care has been actively provided in Korea in various forms, including for inpatients, as a home-based service, and in consultation-based form (Shin et al. 2023). In the Korean context, “hospice palliative care” is an official legal and clinical term defined by this Act, encompassing both hospice and palliative care services for terminally ill patients, regardless of the care setting (Ministry of Government Legislation 2016). This legislation has significantly influenced the approach to end-of-life care, ensuring more comprehensive and accessible palliative care options for terminally ill patients. The Act aims to uphold the dignity and worth of individuals by prioritizing the best interests of patients in the terminal stages of illness by respecting their self-determination (McCormick 2011). This respect for patient self-determination is reflected in legal documents such as an advance directive (AD) or physician orders for life-sustaining treatment (POLST; Kim et al. 2023). In Korea, ADs and POLSTs are distinct both in their legal basis and practical application (Kim and Kim 2023). ADs can be completed by any competent adult in advance, even when they are healthy, and are stored in a national registry. In contrast, POLSTs are clinical orders made jointly by physicians and patients (or their legal proxies) when the patient is nearing the end of life due to a specific life-limiting illness. The POLST is typically used in acute or terminal care settings and has immediate applicability in clinical decision-making. Gradually, many citizens have become interested in hospice care, leading to an increase in its utilization rate (Connor and Bermedo, 2014; Jho et al. 2019; Kwon et al. 2024). To promote the utilization of hospice palliative care for cancer patients, it is essential to provide it alongside curative care from the early stages of treatment (Organization 2014). As part of this effort, Korea introduced hospice palliative care at tertiary hospitals to provide comprehensive palliative care not only in hospice inpatient settings but also for patients being treated in general wards or acute care settings.

Consultation-based hospice palliative care was first introduced as a pilot project in August 2017, and its operation in general hospitals has gradually increased. Placement of a consultation-based hospice palliative care team at hospitals was encouraged by the Korean Ministry of Health and Welfare. Such a team includes doctors, nurses, and social workers who are licensed for consultation-based hospice care by the Korean Ministry of Health and Welfare. The roles of a consultation-based hospice palliative care team are (1) providing information on life-sustaining treatment planning and hospice care and guiding the transfer of patients in need of hospice care to a community hospice setting, (2) delivering advice on hospice care alongside treatment during hospitalization, and (3) coordinating with hospice institutions after discharge or providing post-mortem management. This initiative is expected to increase awareness about self-determination and increase the self-determination respect rate (Kim 2017). Self-determination refers to the right of patients to make informed choices about their own medical care, and the self-determination respect rate is a measure of this practice in medical settings. However, despite the increasing number of consultation-based hospice palliative care teams in tertiary hospitals, no studies have reported either the characteristics of patients referred to these teams or the effect on self-determination respect rate.

This study aimed to describe the characteristics of patients referred to a consultation-based hospice palliative care team and to assess trends in the respect rate of patient self-determination regarding end-of-life care plans, such as AD or POLST, before and after introduction of a consultation-based hospice palliative care team at a tertiary hospital designated as a specialized consultation-based hospice palliative care institution since August 2022. Furthermore, we aim to identify the proportion of deceased patients having end-of-life care plans in the emergency department (ED) or intensive care unit (ICU) of the hospital to ensure that patient wishes are respected in a critical care setting.

## Methods

### Data collection and study subjects

**Figure 1.**
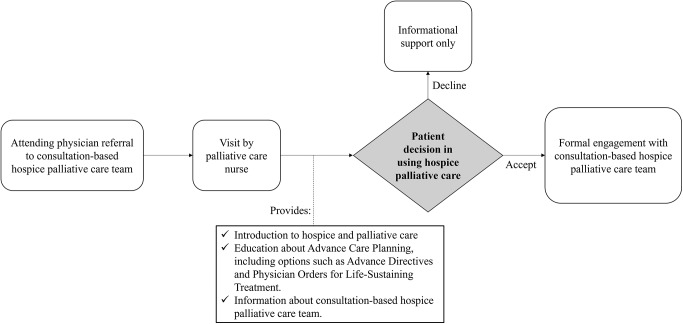
Referral process to consultation-based hospice palliative team.

This study utilized electronic medical records (EMR) and hospice palliative care team data from one of the largest Korean tertiary hospitals from March 2018 to December 2023. In the first part of the investigation, we collected the information of all patients who were referred to the consultation-based hospice palliative care team after its establishment in 2022. All referrals to the consultation-based hospice palliative care team were initiated by the attending physicians based on clinical judgment (Fig. 1). At the time of referral, there was no standardized requirement that goals-of-care discussions be completed prior to the referral. Upon receiving a referral, a palliative care nurse conducted an initial visit with the patient to introduce the concept and goals of palliative care, explain the implications of hospice engagement, and provide education on advance care planning, including options such as AD and POLST. After this initial meeting, patients were given the opportunity to accept or decline further formal palliative care services. Regardless of their decision, all patients received educational support and printed information on palliative care and advance care planning. The diagnosis of cancer was based on ICD-10 codes, and the presence of AD or POLST as documented for patients referred to the consultation-based hospice palliative care team was noted. In the second part, we collected data on the overall number of ADs or POLSTs completed in the hospital, the number of patients who visited the ED or ICU, the number of deaths, and the proportion of deceased patients with AD or POLST in the ED or ICU from March 2018 to December 2023. As ADs can be completed independently by healthy individuals without physician involvement, these are not consistently registered in extractable formats within the EMR. Therefore, the total number of ADs was tracked separately by the hospice palliative care team, who manually updated AD status in the EMR. Since only patients older than 19 years were referred to the team, we selected patients of the same age range who visited the ED or ICU. To minimize bias from retrospective data collection, we used a standardized protocol for data extraction and relied on the hospital’s EMR system, ensuring consistency and completeness. Key variables, such as end-of-life care plan completion, were cross-checked across multiple sources within the EMR to ensure data accuracy.

### Definitions and study outcome

#### Advance care planning: AD or POLST

Advance care planning refers to the structured process through which patients document their decisions about future medical care (Sudore et al. 2017). In Korea, advance care planning primarily involves completing legally recognized documents, such as an AD or POLST (Kim and Kim 2023; Kim et al. 2023). An AD can be completed by any adult, including healthy individuals, to indicate their preferences regarding life-sustaining treatments if they become terminally ill or lose decision-making capacity. The specific contents discussed in completion of an AD are described in Supplementary Table 1. ADs are not limited to the refusal of such treatments but also express a wish to receive all available life-sustaining interventions. The POLST is a medical order designed for patients who are already facing serious, life-limiting illnesses, and it is completed in consultation with a healthcare provider. In the absence of AD or POLST, the patient’s wishes can be presumed based on their usual decisions, and end-of-life medical treatment withdrawal is carried out upon confirmation of consistent statements from two or more family members. In cases where the patient’s wishes cannot be inferred, the consent of all family members is required for implementation (Supplementary Figure xs1).

#### Self-determination respect rates

The main outcome of this study is the trend of patients’ self-determination respect rates before and after introduction of a consultation-based hospice palliative care team at a tertiary hospital. The self-determination respect rate was defined as the proportion of terminally ill patients whose explicit preferences regarding life-sustaining treatment – documented in AD or POLST – were actually respected and implemented in clinical practice. In the Korean context, this metric specifically includes only those patients who chose to withhold or withdraw life-sustaining treatment and whose wishes were honored. Cases where patients requested continuation of life-sustaining therapies, or where decisions were made by family members or surrogates rather than the patient, are not included in this rate. Concordance between documented preferences and actual care was verified through review of EMR, confirming that end-of-life interventions matched the contents of the AD or POLST. This operationalization aligns with the national quality assessment criteria established by the Korean Ministry of Health and Welfare, which uses this metric to monitor the respect for patient autonomy in end-of-life care within healthcare institutions (Go 2024).

#### The ratio of deceased patients with AD or POLST in the ED or ICU

In addition, we identified deceased patients who had completed either an AD or POLST in the ED or ICU. The proportion of this population was calculated by dividing the number of deceased patients with completed AD or POLST by the total number of deceased patients.

#### Statistical analysis

The categorical variables and continuous variables of characteristics of cancer patients referred to consultation-based hospice palliative care team are represented as number (percentage) and mean (standard deviation), respectively. The McNemar test was used to determine whether the completion of AD or POLST was associated with referral to the consultation-based hospice palliative care team. Statistical analysis was conducted using SPSS (version 28.0; IBM Corp., Armonk, NY, USA) with *p* < 0.05 considered statistically significant.

#### Ethnical statement

This study was approved by the Institutional Review Board of Samsung Medical Center (2023-02-021). Informed consent was waived as the study used retrospective medical records without identifying information.

## Results

### Baseline characteristics

Table 1 presents the characteristics of 411 patients referred to the consultation-based hospice palliative care team, of whom 134 (32.6%) agreed to receive continued hospice care. The mean age of those who agreed was 57.9 years, and 72.4% were female. The most common referral departments were Obstetrics and Gynecology (47.8%) and Hematology-Oncology (38.8%).Regarding diagnosis, the largest proportion of referred patients had gynecologic cancers (45.5%), followed by gastrointestinal cancers (20.1%) and hematologic malignancies (17.2%). Detailed diagnostic distributions are provided in Supplementary Table 2.

**Table 1.** Characteristics of patients referred to a consultation-based hospice palliative care team

	Agreement to use consultation-based hospice care service		
	Disagree	Agree	
	N = 277	N = 134	P-value
**Age, years (SD)**	59.6 (13.2)	57.9 (11.5)	0.217
**Female, N (%)**	167 (60.3)	97 (72.4)	0.010
**Referral department, N (%)**			0.385
Obstetrics and Gynecology	91 (32.9)	64 (47.8)	
Haemato-oncology	126 (45.5)	52 (38.8)	
Gastroenterology/ General surgery	34 (12.3)	14 (10.4)	
Neurology/ Neurosurgery	10 (3.6)	0 (0)	
Other departments (Cardiology, intensive unit care, etc.)	16 (5.8)	4 (3.0)	
**Primary diagnosis, N (%)**			0.589
Gynecologic cancers (ovarian, cervical, etc.)	94 (33.9)	61 (45.5)	
Gastrointestinal cancers (gastric, colorectal, hepatobiliary, pancreatic)	56 (20.2)	27 (20.1)	
Hematologic malignancies	41 (14.8)	23 (17.2)	
Lung and thoracic cancers	26 (9.4)	9 (6.7)	
Neurologic diseases/cancers	8 (2.9)	1 (0.7)	
Other solid tumors	28 (10.1)	10 (7.5)	
Rare diseases (amyotrophic lateral sclerosis, amyloidosis, etc.)	7 (2.5)	3 (2.2)	
Unknown/metastasis of unknown origin	1 (0.4)	0 (0.0)	

Abbreviations: SD, standard deviation.

### End-of-life care plan before and after referral to the consultation-based hospice palliative care team

**Figure 2.**
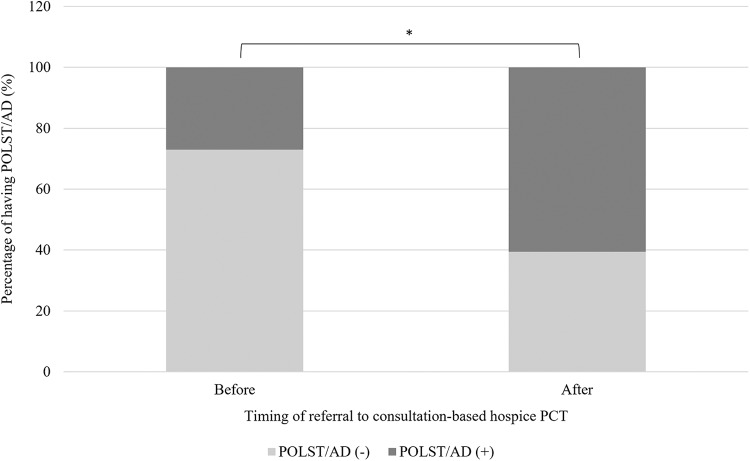
The proportion of patients with completed AD or POLST documents before and after referral to a consultation-based hospice PCT in a tertiary hospital (*N* = 411).

Abbreviations: POLST= Physician Orders for Life-Sustaining Treatment; AD= advance directive; PCT= palliative care team. *p-value < 0.001 is calculated by McNemar test.

The number of patients with documented AD or POLST before referral to a consultation-based hospice palliative care team was 111. After implementation of this team, the proportion increased from 27.0% to 60.6% of a total of 411 patients who were referred to the team and consulted with a hospice nurse (*p*-value < 0.001) (Fig. 2).

### The number of AD or POLST and the self-determination respect rate in a tertiary hospital

**Figure 3.**
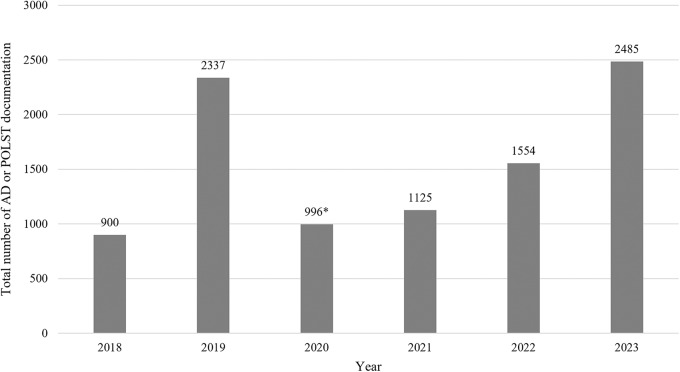
The number of AD or POLST completed in a tertiary hospital from March 2018 to December 2023.

Abbreviations: POLST= Physician Orders for Life-Sustaining Treatment; AD= advance directive. *The number of completed AD or POLST documents increased explosively after enactment of the Life-Sustaining Treatment Decision-making Act in Korea from 2018 to 2019, but decreased in 2020. This significant decrease in nationwide registrations in 2020 is attributed to the COVID-19 pandemic. As the pandemic subsided in the following years, introduction of the consultation-based hospice palliative team in the hospital in 2022 led to a rebound, with increased documentation of AD and POLST orders.

**Figure 4.**
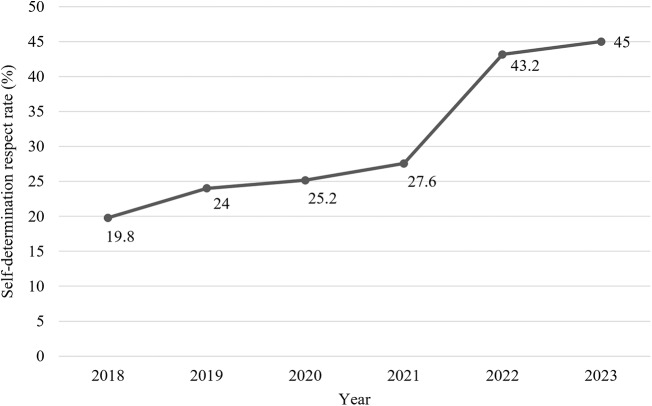
The self-determination respect rate from March 2018 to December 2023 in a tertiary hospital.

The number of AD or POLST documented in a tertiary hospital increased from 2018 and showed a dramatic surge from 2021 to 2023 (*n* = 2485) (Fig. 3). The self-determination respect rate increased every year, from 19.8% in 2018, increasing to 27.6% in 2021, to 43.2% in 2022, and finally to 45% in 2023 (Fig. 4).

### The number of patients with end-of-life care plans who died in either the ED or ICU

**Figure 5.**
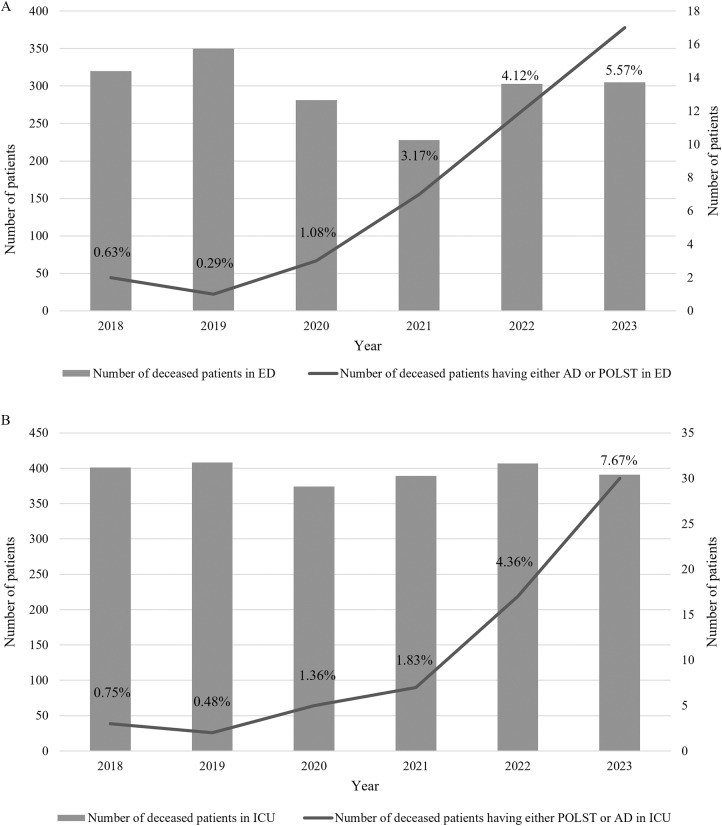
The number of patients with advance care planning (AD or POLST) who died in either the ED (A) or ICU (B) of a tertiary hospital.

Abbreviations: ED= emergency department; ICU= intensive care unit; POLST= Physician Orders for Life-Sustaining Treatment; AD= advance directive. % represents the ratio of deceased patients having either POLST or AD in the ED or ICU.

The number of patients with either AD or POLST who died in either the ED or ICU consistently increased from 2020, with a significant surge from 2021 to 2023 (Fig. 5). The ratio of this population to the total admitted showed a similar pattern, with the highest ratio in 2023 for both the ED and ICU (5.57% and 7.67%, respectively).

## Discussion

We delineated the characteristics of patients referred to our consultation-based hospice palliative care team in a tertiary hospital and showed that the number of documented ADs or POLSTs and the self-determination respect rate increased markedly starting in 2022 when the consultation-based hospice palliative care team was introduced. Furthermore, we demonstrated that referral to the palliative care team promoted the documentation of end-of-life care plans. Additionally, since 2022 when this team was introduced, there was a considerable number of patients with these plans, even in critical care settings like the ED or ICU.

The global perspective on palliative care for terminally ill patients has been gradually shifting toward a more rational approach that emphasizes care since implementation of the Patient Self Determination Act 1991 in the USA (Chakravarty and NK 2002). In Korea, governmental involvement in hospice palliative care began in 2002, with the planning and implementation of a pilot project for hospice palliative care. In 2017, the Life-Sustaining Treatment Decision Act was legislated, and pilot hospice projects were extended to encompass inpatient, home-based, and consultation-based palliative services. Among these measures, the consultation-based hospice service allows patients to receive relevant care from a consultation-based hospice team in general wards or outpatient settings, reimbursed by health insurance. As of September 2024, 42 institutions (31 tertiary hospitals and 11 general hospitals) provide such services. Although hospice-related initiatives in Korea are increasing in use, research on their outcomes and effectiveness remains limited beyond reports published by the Ministry of Health and Welfare.

Most previous studies have highlighted the benefits of palliative care teams in various areas, including economic advantages (cost-saving effects) (May et al. 2015, 2018), symptom management (improved pain relief and quality of life) (Janberidze et al. 2021; Lefkowits et al. 2015), and family outcomes (enhanced family satisfaction) (O’Mahony et al. 2005; Roza et al. 2015). A study conducted in the UK demonstrated that hospital-based palliative care teams could improve cancer patients’ understanding of their disease (Jack et al. 2004). However, the impact of palliative care teams on self-determination regarding end-of-life care planning had not been thoroughly investigated.

To the best of our knowledge, this is the first single tertiary hospital study that reviewed the characteristics of patients referred to a consultation-based hospice palliative care team and reported a subsequent positive outcome in promoting self-determination respect regarding life-sustaining treatment decisions.

### Characteristics of patients referred to the consultation-based hospice palliative care team

Referrals to the consultation-based hospice palliative care team were predominantly for cancer patients. The most common diagnoses were gynecologic, gastrointestinal, and hematologic malignancies. Notably, a substantial portion of referrals came from the Department of Obstetrics and Gynecology, likely due to both the higher prevalence of advanced-stage ovarian cancer and greater departmental engagement with palliative care services. Although standardized hospice education is provided across departments, referral behavior often depends on individual physicians’ awareness and attitudes toward end-of-life care (Mack and Dosa 2020).

### Referral to a consultation-based hospice palliative care team and advance care planning

Our results showed that documentation of end-of-life care plans increased by simply referring patients to the consultation-based hospice palliative care team. Once patients are referred to the team, a hospice nurse visits the patients and provides detailed information about advance care planning and hospice care. One of the reported barriers for patients in making informed decisions and engaging in advance care planning is lack of information (Zalonis and Slota 2014). Therefore, providing timely and appropriate information via a palliative care team to patients with serious illnesses could empower them to exercise their autonomy more effectively for end-of-life care. Our results suggest that the consultation-based hospice palliative care team’s role in providing information on life-sustaining treatment planning and hospice care may have overcome this barrier and promoted end-of-life care planning. It is noteworthy that of the 411 patients referred to the consultation-based hospice palliative care team, only 134 (32.6%) formally agreed to proceed with continued palliative care services. This relatively modest engagement rate likely reflects broader contextual and cultural dynamics specific to Korea. Prior studies have highlighted that many patients in Korean tertiary care settings remain focused on curative treatment goals, often perceiving palliative care as synonymous with imminent death (Baek et al. 2011; Kim et al. 2022, 2023). According to the Korea National Hospice and Palliative Care Registry, only 24.2% of terminally ill cancer patients used hospice services in 2022 (Kim et al. 2024), which is consistent with our observed engagement rate.

Importantly, all referred patients in our study were initially seen by a palliative care nurse, as part of the standard protocol of consultation-based hospice teams in Korea (Kwon and Byun 2024). During this initial encounter, patients were provided with structured information regarding hospice care, AD, and POLST, even if they later declined further palliative involvement. Our findings suggest that even this initial, brief educational interaction played a pivotal role in increasing rates of end-of-life care documentation, regardless of whether patients opted to continue formal hospice care.

### Increase in the number of AD or POLST and the self-determination respect rate

We report the trends of AD and POLST documentation at the tertiary hospital and note that they appear similar to those reported by the Korean Ministry of Health and Welfare (Ministry of Health and Welfare 2020). The numbers of AD and POLST registrations increased after the enactment of the Life-Sustaining Treatment Decision-making Act in Korea, from 2018 to 2019. However, there was a significant decrease in 2020, attributed to a substantial drop in nationwide registrations due to the COVID-19 pandemic (Ministry of Health and Welfare 2020). Starting in 2020, there were annual increases in AD and POLST documentation, culminating in a large peak in 2023. Similarly, the self-determination respect rate showed a trend of increasing after enactment of the Life-Sustaining Treatment Decision-making Act, showing a dramatic surge from 2021 to 2022, when the tertiary hospital introduced the consultation-based hospice palliative care team.

Prior studies have shown that palliative care teams play a crucial role in facilitating end-of-life care discussions and ensuring that patients’ wishes are documented. A retrospective study in the USA reported that inpatient palliative care consultation offered to amyotrophic lateral sclerosis patients resulted in greater documentation of advanced care planning (Mehta et al. 2021). Another study demonstrated that care planning was the most common reason for inpatient palliative care consultation, which in our study is a form of consultation-based hospice palliative care (Bischoff et al. 2018).

Our results align with a prior study in Korea that reported an increase in the rate of self-determination after implementation of the Life-Sustaining Treatment Act and a decrease in the rate of late decisions for completing the end-of-life care plan (Kim et al. 2021). Notably, our study highlighted a significant increase not only in the number of AD and POLST documents completed, but also in the self-determination respect rate in 2022 when the consultation-based hospice palliative care team was introduced in our tertiary hospital.

### Increased number of deceased patients with AD or POLST in critical settings

We observed an increase in the number of patients who completed AD or POLST among those who died in the ED or ICU. Patients in the ED or ICU can also be referred to the consultation-based hospice palliative care team to discuss end-of-life care plans. The purpose of examining this trend was to determine whether introduction of a consultation-based hospice palliative care team would encourage patients in critical settings to design end-of-life care plans. While the ratio of such patients among the total admitted has been increasing since 2018 when the Life-Sustaining Treatment Decision-making Act was enacted, the increase was gradual in the ED but more pronounced in the ICU in 2023, suggesting an impact of the consultation-based hospice palliative care team in a tertiary hospital by promoting ED and ICU patients’ self-determination.

### Clinical implication

Hospice palliative care is recommended to be offered to patients with a life expectancy of 6 months or less (National Cancer Institute 2021). However, in most cases, patients are referred to such care at a very late stage. More than half of the referrals to hospices in UK were reported to occur in the last 7 weeks before death (Allsop et al. 2018), and the mean number of days for referral to hospice care for inpatients in Korea was 24.8 days before death (National Hospice Center 2023). These statistics suggest that most referred patients are in imminent situations (Kim et al. 2021) and could be intimidated to complete end-of-life care plans. Prior studies have investigated the optimal timing for initiating hospice care (Allsop et al. 2018; Petrova et al. 2024) and suggested that routine consultation should be provided early during hospital admission (Castro et al. 2023). Furthermore, the World Health Organization stated that palliative care should be considered from diagnosis onward and integrated into care for people with any condition (Organization 2014). Early referral to hospice can improve symptoms and quality of life, reduce hospital days and aggressive end-of-life treatments for patents, and alleviate caregiver burden and enhance caregiver quality of life (Allsop et al. 2018; Davis et al. 2015; Gaertner et al. 2017). Moreover, early exposure to hospice care, integrated with treatment, can significantly aid patients in seeking self-determination about life-sustaining treatments and better support the projected demand (Allsop et al. 2018). To promote this idea, the role of the consultation-based hospice palliative care team in tertiary hospitals with high admission rates for patients with life sustaining treatments should be highlighted with its role in promoting patient self-determination. Thus, there is a need to encourage introduction of consultation-based hospice palliative care teams to tertiary hospitals.

Even with establishment of consultation-based hospice teams, maximizing positive effects may be challenging without proactive promotion. When patients are referred to the consultation-based hospice palliative care team, they receive information about hospice palliative care and end-of-life care plans from the palliative care team. As a result, our data showed a significant increase in the number of end-of-life care plans after the patients were referred to the palliative care team. Given that attending physicians may find it difficult to provide such information due to their clinical duties, proactive referral to consultation-based hospice could lead to positive outcomes, such as increased rates of end-of-life care plan completion and greater respect for patient self-determination.

### Strengths and limitations

This study has several notable strengths. It explores a critical and underexplored area by examining the impact of consultation-based hospice palliative care teams on self-determination respect rates in end-of-life care, which is essential for patient autonomy. The findings suggest that consultation-based hospice palliative care effectively serves as a bridge, providing crucial information about advance care planning and hospice care to patients in tertiary hospitals. While the research is based in Korea, its implications are far-reaching, offering valuable insights to the international discourse on hospice and palliative care, particularly in addressing the timely provision of care to those in need. However, the single-center, cross-sectional study design limits the ability to establish a causal relationship between introduction of consultation-based hospice care and the observed changes in end-of-life care planning and implementation rates.

## Conclusion

This study demonstrated that introduction of a consultation-based hospice palliative care team enhanced respect for patient self-determination in end-of-life decisions, supporting the integration of such teams in tertiary hospitals to promote early and informed decision-making.

## Supporting information

10.1017/S1478951525100916.sm001 Choi et al. supplementary material 1 Choi et al. supplementary material

10.1017/S1478951525100916.sm002 Choi et al. supplementary material 2 Choi et al. supplementary material

10.1017/S1478951525100916.sm003 Choi et al. supplementary material 3 Choi et al. supplementary material

## Supplementary material

The supplementary material for this article can be found at https://doi.org/10.1017/S1478951525100916.
