# Supplementary material for: Impact of consultation-based hospice palliative care team on self-determination respect rates
Source: Palliat Support Care. 2025 Nov 3;23:e197. doi: 10.1017/S1478951525100916 (PMC13166536; doi:10.1017/S1478951525100916)
Supplement: Choi et al. supplementary material 1 — Choi et al. supplementary material [file S1478951525100916sup001.docx]

**Supplementary Figure 1. Process for Life-Sustaining Treatment Decision-Making**


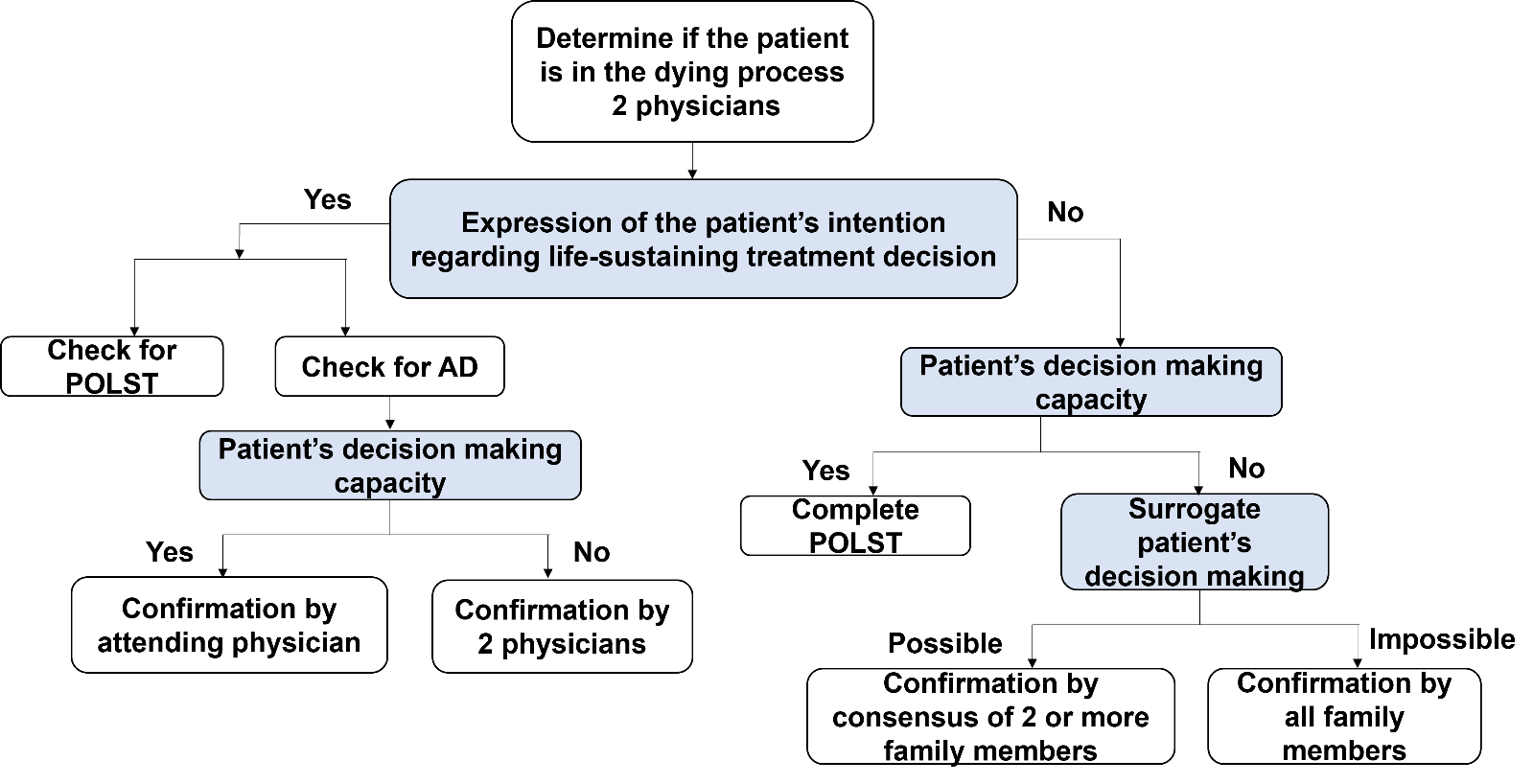
Abbreviations: POLST, Physician Orders for Life-Sustaining Treatment; AD, advance directive.
