# Supplementary material for: Impact of consultation-based hospice palliative care team on self-determination respect rates
Source: Palliat Support Care. 2025 Nov 3;23:e197. doi: 10.1017/S1478951525100916 (PMC13166536; doi:10.1017/S1478951525100916)
Supplement: Choi et al. supplementary material 2 — Choi et al. supplementary material [file S1478951525100916sup002.docx]

**Supplementary Table 1. Contents discussed in completion of Advance Directives in South Korea**

| **Category** | **Details** |
| --- | --- |
| **Decision on Life-Sustaining Treatment (LST)** | Individuals specify whether they wish to withhold or withdraw LST such as cardiopulmonary resuscitation (CPR), mechanical ventilation, hemodialysis, or anticancer therapy at the end of life. |
| **Hospice and Palliative Care** | Individuals may choose whether to receive hospice care in lieu of aggressive life-sustaining measures. |
| **Validity and Legal Status** | Information is provided on the legal effect, duration, and conditions that may render the directive invalid. The AD becomes effective only when the patient reaches the end-of-life stage and is unable to make decisions. |
| **Registration and Recordkeeping** | Details include how to prepare, register, store, and notify others about the existence of an AD. The directive must be registered with an official agency approved by the Ministry of Health and Welfare. |
| **Revision and Revocation** | Individuals may amend or revoke their AD at any time. The process for doing so and the legal consequences of changes are explained. |
| **Handling of Records** | In the event of the closure, suspension, or de-designation of an AD registry agency, procedures are in place to ensure the secure transfer of records. |
| **Educational Requirements** | Completion of an AD must be preceded by standardized education from trained personnel to ensure understanding of the legal, medical, and ethical implications. |
| **Voluntariness and Informed Consent** | The directive must be completed voluntarily without coercion, and only after the individual has been fully informed of the options and consequences. |
