# Supplementary material for: Impact of consultation-based hospice palliative care team on self-determination respect rates
Source: Palliat Support Care. 2025 Nov 3;23:e197. doi: 10.1017/S1478951525100916 (PMC13166536; doi:10.1017/S1478951525100916)
Supplement: Choi et al. supplementary material 3 — Choi et al. supplementary material [file S1478951525100916sup003.docx]

**Supplementary Table 2. Detailed diagnostic distributions in patients referred to a consultation-based hospice palliative care team**

|  | **Agreement to use consultation-based hospice care service** | |  |
| --- | --- | --- | --- |
|  | **Disagree** | **Agree** | **P-value** |
|  | N=277 | N=134 |  |
| **Diagnosis** |  |  | 0.589 |
| Ovarian cancer | 46 (16.6) | 32 (23.9) |  |
| Hematologic cancer | 41 (14.8) | 23 (17.2) |  |
| Gastrointestinal cancer | 28 (10.1) | 10 (7.5) |  |
| Lung cancer | 24 (8.7) | 8 (6.0) |  |
| Cervical cancer | 21 (7.6) | 16 (11.9) |  |
| Hepatocarcinoma | 18 (6.5) | 8 (6.0) |  |
| Pancreatic cancer | 18 (6.5) | 7 (5.2) |  |
| Endometrial cancer | 16 (5.8) | 9 (6.7) |  |
| Sarcoma | 10 (3.6) | 1 (0.7) |  |
| Uterine cancer | 9 (3.2) | 3 (2.2) |  |
| Central nervous system cancer | 7 (2.5) | 1 (0.7) |  |
| Cholangiocarcinoma | 6 (2.2) | 2 (1.5) |  |
| Breast cancer | 4 (1.4) | 5 (3.7) |  |
| Bladder cancer | 4 (1.4) | 5 (3.7) |  |
| Peritoneal cancer | 3 (1.1) | 1 (0.7) |  |
| Oropharyngeal cancer | 3 (1.1) | 1 (0.7) |  |
| Prostate cancer | 2 (0.7) | 1 (0.7) |  |
| Interstitial lung disease | 2 (0.7) | 0 (0) |  |
| Malignant thymoma | 2 (0.7) | 0 (0) |  |
| Vulvar cancer | 2 (0.7) | 0 (0) |  |
| Gallbladder cancer | 1 (0.4) | 1 (0.7) |  |
| Tongue cancer | 0 (0) | 1 (0.2) |  |
| Malignancy of pleural effusion | 0 (0) | 1 (1.4) |  |
| Amyloidosis | 0 (0) | 1 (0.7) |  |
| Amyotrophic lateral sclerosis | 1 (0.4) | 0 (0) |  |
| Spinal cancer | 1 (0.4) | 0 (0) |  |
| Metastasis of unknown origin | 1 (0.4) | 0 (0) |  |
| Retroperitoneal cancer | 1 (0.4) | 0 (0) |  |

Values presented as numbers (%)
